# Self-Lubricating and Shape-Stable Phase-Change Materials Based on Epoxy Resin and Vegetable Oils

**DOI:** 10.3390/polym15194026

**Published:** 2023-10-09

**Authors:** Svetlana O. Ilyina, Irina Y. Gorbunova, Veronika V. Makarova, Michael L. Kerber, Sergey O. Ilyin

**Affiliations:** 1 A.V. Topchiev Institute of Petrochemical Synthesis, Russian Academy of Sciences, 29 Leninsky Prospect, 119991 Moscow, Russia; 2 Department of Plastics Processing Technology, D. Mendeleev University of Chemical Technology of Russia, 9 Miusskaya Square, 125047 Moscow, Russia

**Keywords:** phase-change materials, epoxy resin, vegetable oil, miscibility, rheology, thermophysical properties, friction, self-lubrication

## Abstract

Palm or coconut oil is capable of dissolving in a mixture of bisphenol A-based epoxy resin and a high-temperature hardener (4,4′-diaminodiphenyl sulfone) when heated and then forms a dispersed phase as a result of cross-linking and molecular weight growth of the epoxy medium. Achieving the temporary miscibility between the curing epoxy matrix and the vegetable oil allows a uniform distribution of vegetable oil droplets in the epoxy medium. This novel approach to creating a dispersed phase-change material made a cured epoxy polymer containing up to 20% oil. The miscibility of epoxy resin and oil was studied by laser interferometry, and phase state diagrams of binary mixtures were calculated according to theory and experiments. A weak effect of oil on the viscosity and kinetics of the epoxy resin curing was demonstrated by rotational rheometry. According to differential scanning calorimetry and dynamic mechanical analysis, the oil plasticizes the epoxy matrix slightly, expanding its glass transition region towards low temperatures and reducing its elastic modulus. In the cured epoxy matrix, oil droplets have a diameter of 3–14 µm and are incapable of complete crystallization due to their multi-component chemical composition and non-disappeared limited miscibility. The obtained phase-change materials have relatively low specific energy capacity but can be used alternatively as self-lubricating low-noise materials due to dispersed oil, high stiffness, and reduced friction coefficient. Palm oil crystallizes more readily, better matching the creation of phase-change materials, whereas coconut oil crystallization is more suppressed, making it better for reducing the friction coefficient of the oil-containing material.

## 1. Introduction

The storage of thermal energy is a significant aspect of power supply management [1,2,3]. The operating principle of heat accumulators is to use sensible, latent, or chemical thermal energy [4]. In addition to storing energy, thermal accumulators can provide uninterrupted operation of heating systems in both residential and industrial buildings, storing heat during the day and releasing it at night [5]. The most effective basis for creating accumulators is phase-change materials (PCMs) that use latent heat energy. They absorb or release heat during the transition between liquid and solid aggregate states [6]. The advantage of PCMs is that the amount of absorbed or released energy due to their melting or crystallization is much higher than that from their heat capacity and increases or decreases in temperature [7,8,9].

PCMs are divided conditionally into organic, inorganic, and eutectic materials. Organic PCMs can be from paraffin waxes, glycols, fatty acids, fatty alcohols, and their esters [10]. The advantages of organic PCMs are their ability to absorb and release large amounts of heat, to tolerate multiple heating and cooling cycles, not to separate into phases, and to crystallize without strong supercooling [11]. However, these materials have disadvantages, such as high flammability and low thermal conductivity [10,11,12,13], which can be overcome by obtaining composite PCMs using various solid particles [14,15]. Inorganic PCMs represent salt hydrates, low-melting metals, or metal alloys [16]. Compared with organic PCMs, inorganic salts have a higher thermal capacity, while low-melting metals have excellent thermal conductivity. These materials have found application in solar energy systems, thermal systems of buildings and other constructions, and mechanical engineering [17]. The disadvantages of inorganic materials are the low corrosion resistance of metals, the relatively low thermal conductivity of molten salts, the tendency to supercool, and the thermal instability of salt hydrates [18]. Eutectic materials are homogeneous mixtures of two or more components. Mixed, they melt (or crystallize) wholly at a single temperature, which, in turn, is lower than the melting temperatures of the components taken separately. The advantage of eutectic materials is the ability to combine both organic and inorganic ingredients. As a result, there are more opportunities to obtain formulations with the required properties. The most common organic constituents for eutectic mixtures are fatty acids, such as capric, myristic, lauric, stearic, or palmitic, while salt hydrates usually act as their inorganic complementary components [10,19,20].

Great attention is paid to giving PCMs shape stability by using various polymer materials in their composition [21]. Polymers can act as PCMs, serve as the basis for obtaining composites with a phase-changeable dispersed phase, or be materials for creating capsules with substances that store energy [22]. Encapsulation prevents leakage of phase-change substances in their liquid state during heating and cooling cycles and isolates them from the influence of the environment [23,24]. It solves the problem of corrosiveness and ignition hazard of inorganic and organic constituents of PCMs, respectively. In addition, encapsulation makes it possible to reduce the negative effect of changes in PCM volume during the phase transition and improve its thermal conductivity and heat storage efficiency [25,26]. The most promising methods are micro- and nanoencapsulation [20,27,28,29], and in situ polymerization is the most appropriate method for achieving the structural integrity of the resulting capsules [30]. The disadvantages of encapsulation are the increased technological complexity of obtaining the capsules and the lower specific energy efficiency of PCMs due to the unused volume between them.

The application of continuous polymer matrices is also common to ensure the shape stability of PCMs, as this manufacturing method is more practical and less costly than encapsulation [31]. Polyethylene, polypropylene, polyoxymethylene, and polycaprolactam are most commonly used as polymer matrices because they have high heat capacity and good thermal and thermo-oxidative stability [32]. However, the disadvantages of thermoplastic polymers are their high viscosity and the need for high-temperature mixing with a phase-change substance that is in the liquid state and has a much lower viscosity due to its low-molecular-weight nature. In turn, a significant difference in the viscosities of the compounding components leads to a high value of the critical capillary number, which should be achieved to disperse the PCM droplets in the volume of the polymer matrix [33,34]. At the same time, a high capillary number requires a high shear rate, which is not realizable in practice due to the high viscosity of the polymer melt and its transition into a forced rubbery-like state with a loss of steady-state fluidity [35,36]. For this reason, only coarse dispersions with a low content of the dispersed phase can be formed based on thermoplastic polymers. The solution to this problem may be to apply low-viscosity thermosetting binders or high-temperature miscible PCM/elastomer blends [37]. Thermosetting binders are available in different variants, letting them be tailored to any specific application. From the rheological point of view, they can allow for better dispersing phase-change substances both in a liquid state (due to the low difference in viscosities of continuous and dispersed phases) and in a solid one (owing to the low viscosity of the thermosetting polymer).

Epoxy polymers have low shrinkage, good strength, thermal stability, solvent resistance, and ease of fabrication [38,39]. Their curing parameters can be easily varied by selecting a hardener [40], which reacts with the epoxy groups of the resin to form a three-dimensional cross-linked structure, where a PCM can be reliably enclosed. Because of the spatial network of covalent bonds, the cured composite is stiff and able to keep a constant shape, which excludes the probability of leakage of the dispersed phase in its liquid state. In general, shape-stabilized PCM–epoxy composites are used for thermal energy management in buildings and constructions, and their preparation techniques include blending, encapsulation, impregnation, and lamination methods [41]. Previously, epoxy phase-change materials were based on the expanded graphite/paraffin composite sealed with cured epoxy resin [38,42], epoxy/polyethylene glycol blends containing expanded graphite and Ag nanoparticles [43], epoxy/paraffin emulsions stabilized by asphaltenes [44], graphite/epoxy encapsulating scaffold containing graphite-filled paraffin [45], epoxy phosphaphenanthrene containing supporting materials for encapsulated polyethylene glycol [46], paraffin encapsulated in crystallizable epoxy resin cured with poly(propylene oxide)diamine [47], and cured allyl-based epoxy resin acted as phase-change materials due to its crystallizable 1-octadecanethiol grafted groups [31]. In other words, quite complex and expensive techniques and compositions are used for obtaining epoxy PCMs, making the search for easier ones a relevant challenge.

Due to its small molecular weight, epoxy resin demonstrates miscibility with many low- and high-molecular-weight substances, and this miscibility often disappears during the curing because of an increase in the molecular weight [48,49]. This feature opens up the possibility of using a phase-change agent that is temporarily miscible with the epoxy resin under curing conditions but forms a dispersed phase because of the phase separation caused by an increase in the molecular weight of the reaction system with time. In this case, one can expect the formation of finely dispersed phase-change droplets resistant to coalescence thanks to the high or even infinite viscosity of the epoxy resin at the point of phase separation [50]. An organic substance close in polarity to epoxy resin should be taken as a miscible phase-change agent. Paraffin wax is non-polar and insoluble in epoxy resins [44], while crystallizable glycols have strong intermolecular hydrogen bonds that make them immiscible with conventional bisphenol A-based epoxy resins. These facts limit the choice of PCMs to fatty acids, fatty alcohols, and their esters. In this case, we can use natural raw materials: vegetable oils that are mixtures of triglycerides from fatty acids [51]. Since miscibility usually increases with rising temperature (at least in the absence of hydrogen bonds [52,53,54,55]), it is reasonable to perform the mixing of an epoxy resin, a hardener, and a phase-change agent at a high temperature for better mutual solubility. In turn, this limits the range of possible hardeners to low reactive substances to prevent rapid cross-linking of the system with the resulting high internal stresses. On this basis, the work aimed to obtain and then study blends of epoxy resin, high-temperature hardener, and high-melting vegetable oils to evaluate their potential to be a shape-stable phase-change material.

## 2. Materials and Methods

### 2.1. Materials

The thermosetting binder was diglycidyl ether of bisphenol A (D.E.R. 330, Dow Chemical, Midland, MI, USA) containing 180.5 g/mol-eq epoxy groups. The hardener was 4,4′-diaminodiphenyl sulfone (DDS, Sigma-Aldrich, Steinheim, Germany) with an amine group content of 62.1 g/mol-eq. The weight ratio of epoxy resin to hardener was stoichiometric (74.4/25.6 wt.%/wt.%).

High melting point vegetable oils were used as PCMs: refined palm oil from Malaysia (*T*_m_ = 36 °C) and refined, bleached, and deodorized coconut oil from Thailand (*T*_m_ = 21 °C), which were purchased at a local market. Palm oil contains mostly triglycerides of oleic and palmitic acids [56], while coconut oil belongs to the lauric group of vegetable oils and differs by its sharp transition from solid to liquid states within a narrow temperature range [57].

Blends of epoxy resin, hardener, and one of the vegetable oils were prepared by mechanical mixing on a magnetic stirrer at 60 °C. The oil content in the resin/hardener mixture was 0, 5, 10, or 20 wt.%. The blends were cured at 180 °C for 3 h.

### 2.2. Methods

The miscibility of epoxy resin with vegetable oils was evaluated by laser interferometry according to the method described in detail earlier [58,59]. The diffusion cell with samples was subjected to stepwise heating from 20 °C to 110 °C followed by slow cooling to 30 °C. Higher temperatures were not used since the experiment was not workable because of the strong interface curvature caused by the stirring of the resin and the oil due to their low viscosities. Microphotographs of diffusion zones were obtained using an objective with 3.5× magnification and a digital camera with a 12 Mpx 1/1.7″-type IMX226 CMOS image sensor (Sony, Tokyo, Japan). A diode laser KLM-A532-15-5 (FTI-Optronic, Saint Petersburg, Russia) with a wavelength of 532 nm was used as the light source.

A study of rheological properties was carried out on a Discovery HR-2 rotational rheometer (TA Instruments, New Castle, DE, USA). Flow curves and frequency dependences of storage and loss moduli of uncured samples were obtained at 25 °C using a cone–plate measuring unit with a cone/plate angle of 2° and a cone base diameter of 40 mm. Flow curves were obtained by increasing the shear rate from 10^−4^ to 10^3^ s^−1^ in a stepwise mode with a test time for each shear rate value of 60 s. Viscoelastic properties were studied at a strain amplitude of 0.1% in an angular frequency range of 0.0628–628 rad/s. To investigate the kinetics of sample curing, a plate/plate unit was used with a plate diameter of 8 mm and a distance between the plates of 500 µm. In the latter case, temperature dependences of effective viscosity were obtained at a heating rate of 2 °C/min and a shear rate of 100 s^−1^. Tests were performed two or three times to confirm the reproducibility of the obtained data. Rheological characteristics were calculated using standard equations [60,61], and the relative error in their measurement was no more than 5%.

Differential scanning calorimetry (DSC) was performed on a DSC823e calorimeter (Mettler Toledo, Columbus, OH, USA) in an argon medium. Measurements of the heat flow emitted during the curing of uncured samples were carried out in the heating mode at a rate of 2 °C/min in the temperature range 100–280 °C. The slow heating rate provides sufficient time for curing without severe temperature rise and thermal degradation. Thermograms of the pre-cured samples were obtained from −50 °C to 200 °C at a temperature rise of 10 °C/min. Fast heating was for a sharp change in heat capacity due to the glass/rubber transition and its higher sharpness on DSC curves. Temperature scans were repeated at least 2–3 times for samples weighing 10–15 mg to confirm their reproducibility and calculate average values. The accuracy of determination of the transition temperatures was ±0.2 °C, while the relative error of evaluation of their enthalpies did not exceed 5%.

The viscoelastic properties of cured blends were investigated via dynamic mechanical analysis (DMA) on the same Discovery HR-2 rheometer by two-point bending using a measuring unit with a single cantilever clamp at a deformation frequency of 1 Hz, a relative strain amplitude of 0.01%, and a temperature rise rate of 5 °C/min in the range 20–240 °C. The cured samples had a length of 16 mm, a width of 10.5 mm, and a height of 2.3–3.0 mm. Number of replications of each test was 2 to 3 times.

Microphotographs of samples between two coverslips before and after their curing were taken using a 10× magnification lens on a digital camera with a Sony IMX226 sensor (1/1.7″, 12 Mpx).

Scanning electron microscopy (SEM) of fracture surfaces of cured samples was performed on a Phenom XL G2 microscope (Thermo Fisher Scientific, Eindhoven, The Netherlands) at 15 kV accelerating voltage and 60 Pa pressure. To remove the electric charge, a thin (about 5 nm) layer of gold was preliminarily applied to the surfaces by ion-plasma sputtering on the Cressington 108auto sputter coater (Cressington Scientific Instruments, Watford, UK).

Tribological tests were performed on the above-mentioned Discovery HR-2 rheometer using a plate–plate friction pair where a steel plate with a diameter of 8 mm rotated on a second plate made of the tested material with an angular velocity of 100 rad/s and an axial force *F*_Z_ = 30 N at 25 °C. The coefficient of friction was calculated using the following formula:

(1) $$\mu =\frac{3M}{2RF_{Z}},$$

where *M* is the torque of the rheometer shaft, while *R* is the radius of the steel plate. For any formulation under study, the measurements were repeated at least four times for 10 min per test with a subsequent substitute of a plate from the tested material after each run. Due to the high angular velocity, the specified test time was sufficient for lapping the moving plates and reaching the steady-state value of the friction coefficient, whereas lengthier friction caused substantial heating of the steel plate and thus was inappropriate. Because of the tests, the wear of the material under examination was negligible for the measurement. Before each test, we degreased plates with acetone.

## 3. Results and Discussion

### 3.1. Miscibility of Epoxy Resin and Vegetable Oils

**Figure 1:**
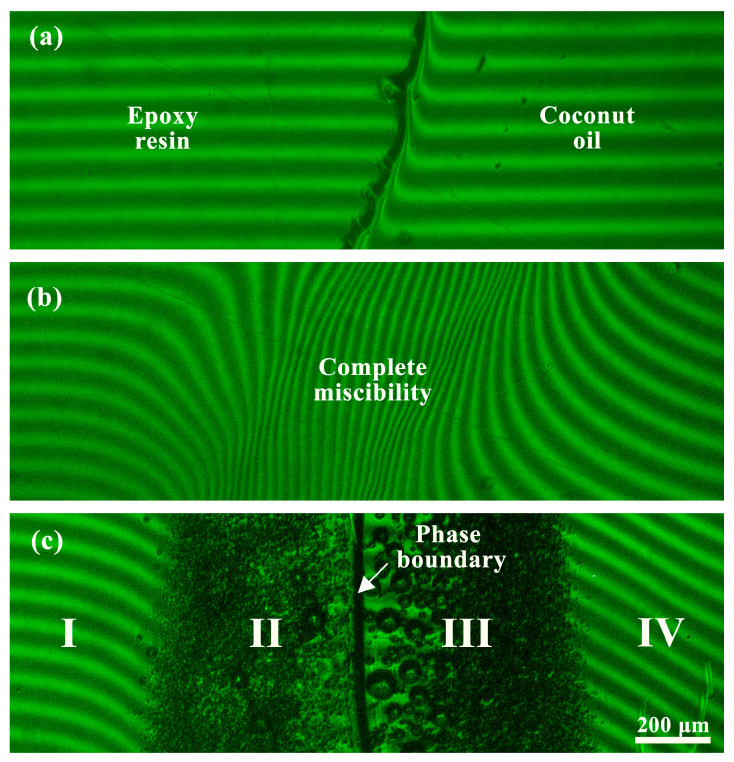
Interferograms of mutual diffusion zones of epoxy resin (left) and coconut oil (right) at 30 °C (**a**,**c**) and 100 °C (**b**) for systems without temperature history (**a**,**b**) and after preheating to 100 °C (**c**).

Figure 1 shows interferograms of the mutual diffusion zone of epoxy resin and coconut oil. According to the obtained data, there is a pronounced interface at 30 °C (Figure 1a), indicating no complete mutual solubility (miscibility) between epoxy resin and coconut oil. However, there are distortions of the bands on the side of the vegetable oil phase on the interferogram. This fact suggests that the epoxy resin diffuses into the oil phase. However, there are no distortions of the bands on the resin side, which demonstrates the inability of the oil to dissolve appreciably in the resin medium under this temperature.

Upon heating, the curvature of the interference bands increases, curvatures appear on the resin side as well, and the phase boundary disappears at 100 °C (Figure 1b), i.e., the resin and the oil become indefinitely mutually soluble. When the diffusion cell is cooled, liquid–liquid phase separation occurs: the phase boundary reappears with the formation of emulsion droplets on either side of it (Figure 1c). As a result, the contact of epoxy resin and coconut oil produces four concentration zones: oil solution in resin (Figure 1c, zone I), oil emulsion in saturated oil solution in resin (II), resin emulsion in saturated resin solution in oil (III), and resin solution in oil (IV).

**Figure 2:**
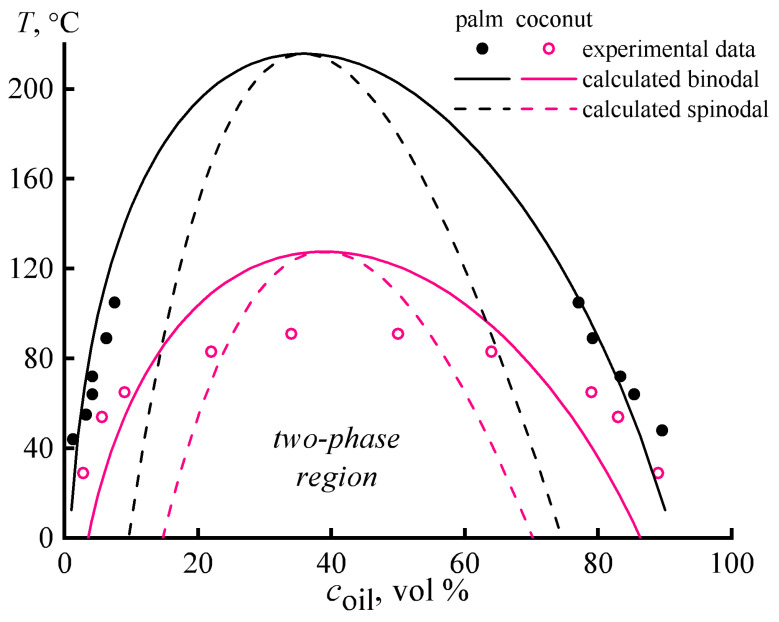
Phase diagrams for epoxy resin blends with palm oil or coconut oil. The points indicate the experimental results, while the lines represent the calculated binodal and spinodal curves.

In the case of palm oil, the interferograms of the diffusion zones are generally similar, except that there is no complete miscibility, at least upon heating to 110 °C. Nevertheless, the obtained interferograms in the considered temperature region 30–110 °C allow for calculating fragments of phase diagrams for mixtures of epoxy resin with palm and coconut oils (Figure 2, points).

Phase diagrams can also be calculated theoretically based on the condition of equality of the free energy increments for each of the system components in the coexisting phases [62,63,64]:

(2) $$\Delta \bar{G_{i}^{\prime }}=\Delta \bar{G_{i}^{\prime \prime }},$$

where $\Delta \bar{G_{i}^{\prime }}$ and $\Delta \bar{G_{i}^{\prime \prime }}$ are the increments of the partial molar free energy of the *i*-th component in the first and second phases, respectively. In the case of a two-component mixture, the incremental partial molar free energy can be calculated using the following equation:

(3) $$\Delta \bar{G_{1}^{\prime }}=RT(ln\varphi_{1}+\frac{V_{m,2}-V_{m,1}}{V_{m,2}}\varphi_{2}+\chi_{12}\varphi_{2}^{2}),$$

where *φ*_1_ and *φ*_2_ are the volume fractions of the first and second components, respectively, *V*_m,1_ and *V*_m,2_ are their molar volumes, *R* is the universal gas constant, *T* is the thermodynamic temperature, and *χ*_12_ is the Flory–Huggins interaction parameter. For the second component, the form of the equation does not change (except for the corresponding indices).

The Flory–Huggins interaction parameter can be calculated from the Hildebrand solubility parameters of the interacting mixture components:

(4) $$\chi_{12}=\chi^{s}+\frac{V_{m,1}{\left(\delta_{1}-\delta_{2}\right)}^{2}}{RT},$$

where *χ*^s^ is an entropic correction factor that takes values of 0 or 0.25 for low- or high-molecular-weight systems, respectively, while *δ*_1_ and *δ*_2_ are the Hildebrand solubility parameters for the first and second components.

The Hildebrand solubility parameter of some substances is diectly related to its enthalpy of evaporation Δ*H*_v_ [65]:

*δ*^2^ = (Δ*H*_v_ − *RT*)/*V*_m_. (5)

For the epoxy resin, the solubility parameter is 21.56 MPa^0.5^ [66,67], while the molar volume is 290.6 cm^3^·mol^−1^. Coconut and palm oils are mixtures of triglycerides, i.e., they consist of substances with different solubility parameters and molar volumes. However, coconut oil contains mainly glyceryl trilaurate, while palm oil consists predominantly of glyceryl tripalmitate [56,57]. In turn, the solubility parameters and molar volumes of glyceryl trilaurate (17.64 MPa^0.5^ and 714.79 cm^3^·mol^−1^ [68]) and glyceryl tripalmitate (17.44 MPa^0.5^ and 932.27 cm^3^·mol^−1^) allow using Equations (2)–(5) to calculate the binodal lines in first approximation (Figure 2, solid lines), assuming that *V*_m_ and *δ* for oils and epoxy resin change equally with temperature.

In addition, it is possible to calculate spinodal lines for the same systems, where the second derivative of the free energy on the concentrations of the mixture components takes a zero value [69]:

(6) $$\frac{d^{2}\Delta G}{d\varphi_{1}d\varphi_{2}}=0.$$

As applied to a two-component mixture, the Flory–Huggins equation for free energy can be expressed as follows [70]:

(7) $$\Delta G=RT\left(\frac{\varphi_{1}}{V_{m,1}}ln\varphi_{1}+\frac{\varphi_{2}}{V_{m,2}}ln\varphi_{2}+\chi_{12}\varphi_{1}\varphi_{2}\right).$$

Then, taking the Equations (4) and (6) into account, the following expression for the spinodal lines can be obtained:

(8) $$\frac{1}{{V_{m,1}\varphi }_{1}}+\frac{1}{{V_{m,2}\varphi }_{2}}-2(\frac{{\left(\delta_{2}-\delta_{1}\right)}^{2}}{RT})=0$$

the result of its application is shown by the dashed lines in Figure 2.

The calculated and experimental phase diagrams are needed to predict the phase behavior of blends at high temperatures before their curing, as the phase behavior determines the possibility of curing with the preservation of the macro-homogeneous structure of these blends. If they are emulsions in the hot state, they will be unstable and separated into layers since we do not use surfactants. Therefore, homogeneous hot blends are needed to start the curing, and the phase diagrams can provide their compositions. Although further curing increases the molecular weight of epoxy resin, changes the phase diagrams, and impairs miscibility, the resulting phase separation is not a problem due to the significantly increased viscosity of the blends.

In the case of palm oil, the experimental points overlap well with the binodal lines found by the calculation method. The calculated upper critical solution temperature (UCST) for the palm oil/epoxy resin system is 216 °C, i.e., significantly higher than the temperature used for curing epoxy compositions (180 °C). In other words, complete miscibility between palm oil and epoxy resin is impossible, even under high-temperature curing conditions. Meanwhile, the maximum solubility of the oil in the resin at the curing temperature is about 16 vol.% (14.3 wt.%). Note that under the same temperature, the solubility of the epoxy resin in the oil is more than twice as high (40 vol.%).

Coconut oil dissolves better in epoxy resin than palm oil. Complete miscibility with epoxy resin happens at about 90–120 °C. At the same time, there is a discrepancy between the experimental and calculated data. The UCST determined experimentally turns out to be 30 °C lower, with binodals that are less intensely temperature-dependent. The broader region of the two-phase state and the lower value of the UCST of the actual system are likely due to the multi-component composition of the oil, which includes both lower and higher molecular weight homologs of glyceryl trilaurate. Furthermore, the UCST is below the curing temperature of the resin, indicating that coconut oil is better suited to produce a homogeneous mixture. However, the position of the UCST on the concentration axis corresponds to the coconut oil content of 38 vol.% (24.4 wt.%). This fact means there is no point in introducing more than 24 wt.% of coconut oil into the resin, as a higher oil concentration results in an emulsion of epoxy resin in the oil, which turns into a suspension of epoxy polymer particles during the curing. However, the solubility of the oil in the resin is only about 2.5 wt.% at 25 °C, although the solubility of the resin in the oil is up to 11 wt.% at this temperature.

Thus, mixtures of epoxy resin and coconut oil are emulsions under normal temperature conditions due to the immiscibility and liquid state of this oil, whereas mixtures of the resin and palm oil are suspensions because of the immiscibility and semi-solid state of the oil. Moreover, if a powdered hardener is added to the mixture, the composition of the dispersions becomes even more complex. In the case of coconut oil, it is mixtures including solid hardener particles and liquid oil droplets, while dispersions of palm oil include two types of solid particles. It can be expected that the complex composition of dispersions has a significant impact on their rheological properties.

### 3.2. Rheology of Uncured Blends

**Figure 3:**
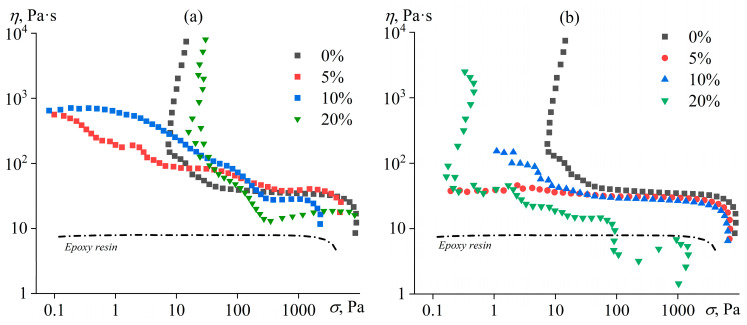
Dependences of effective viscosity on shear stress for epoxy resin/hardener blends containing palm oil (**a**) or coconut oil (**b**) at 25 °C. The oil concentration is given in the legend.

The viscosity (*η*) of the pure resin is constant at low to moderate shear stresses (*σ*) but decreases slightly at stresses above 2000 Pa (Figure 3). Typically, the decrease in viscosity in polymer systems is attributed to the orientation of entangled macromolecules at high shear rates. The degree of polymerization of individual epoxy resin molecules does not exceed 3–4, i.e., epoxy resin has no entanglements and, accordingly, cannot enter the forced rubbery state with disentanglement and orientation of macromolecular chains, leading to a decrease in effective viscosity [71]. In this regard, the reduction in viscosity of the resin can be due to its gradual transition to the forced glassy state under the action of high shear rates [72]. Such a transition occurs when the test temperature is not much higher than the glass transition temperature of the material; for example, similar non-Newtonian behavior is exhibited by glass-forming liquids without macromolecular entanglements, such as heavy crude oils, bitumens, and solutions and melts of asphaltenes [73,74].

The introduction of a hardener into the resin leads to the appearance of the yield stress—a narrow stress region of about 10 Pa, within which the effective viscosity drops by several decimal orders of magnitude (Figure 3). A percolation structure is in the system due to the mutual interaction of hardener particles, and its strength is equal to the yield stress [75,76]. However, even under high stresses that destroy the structural network of hardener particles, the viscosity of the resin/hardener mixture is almost five times higher than the viscosity of the pure resin. This fact means a strong interaction between the hardener particles even under flow conditions.

The effect of both types of vegetable oil on the viscosity properties of the resin/hardener blends is quite similar. The addition of 5–10% oil causes a change in the flow curve, manifested by the disappearance of the yield stress. Perhaps the yield stress disappearance is due to the surface activity of the oil, which is adsorbed on the surface of the hardener particles and thus reduces the interfacial tension [77]. In turn, surfactant adsorption suppresses interactions between the particles and reduces the yield stress [34]. In the case of coconut oil, the viscosity decreases in the whole range of shear stresses, which can be due to the dissolution of the oil as a less viscous liquid in the epoxy medium (Figure 3b). For 5% coconut oil, the blend even acquires a Newtonian behavior, indicating the deagglomeration of the solid particles and the absence of their interaction within the epoxy continuous medium. In contrast, mixtures containing 5–10% palm oil have a pseudoplastic behavior, probably caused by the disagglomeration of solid particles when the shear stress increases (Figure 3a). Since palm oil has a semi-solid consistency, an increase in its mass fraction from 5% to 10% raises the number of solid particles and thus elevates the viscosity in the low-stress region when the particles agglomerate. High shear stresses break the agglomerates and lower the viscosity, which becomes even lower in the case of 10% coconut oil, probably due to the oil’s partial solubility in the epoxy medium.

At 20% concentrations of oils, the yield stress appears again, probably due to the formation of a new structure of interacting oil droplets [78,79,80,81]. In addition, one can observe a significant decrease in effective viscosity at high shear stresses, which may be due to the wall slip of the blends on the measuring surface of the rheometer units [82,83,84,85]. A more substantial wall slip (expressed as a more pronounced drop in effective viscosity) occurs with coconut oil, which is in the liquid state at the test temperature. Meanwhile, 20% palm oil provides higher yield stress and high-shear viscosity than 20% coconut oil due to its semi-solid consistency and the fact that the viscosity and yield stress of suspensions are significantly higher than those of emulsions with the identical content of dispersed solid particles or droplets [83,86].

**Figure 4:**
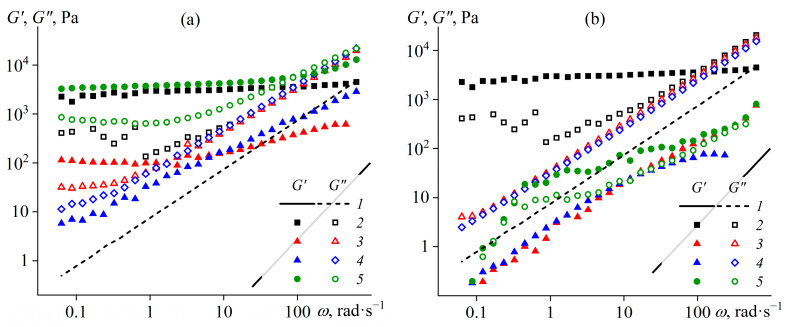
Frequency dependences of storage and loss moduli for epoxy resin (*1*) and epoxy resin/hardener mixtures (*2*–*5*) containing palm oil (**a**) or coconut oil (**b**) at 25 °C. The oil concentration was 0 (*2*), 5 (*3*), 10 (*4*), or 20 (*5*) wt.%.

A study of the viscoelastic properties of epoxy compositions can provide additional information about their structure. Frequency dependences of storage and loss moduli for blends containing different concentrations of palm or coconut oil are shown in Figure 4. Pure epoxy resin is a Maxwell fluid whose loss modulus (*G*″) grows linearly with increasing angular frequency (*ω*) while the storage modulus (*G*′) rises twice as intensely. The addition of the hardener into the epoxy resin causes its structuring. Firstly, the storage modulus becomes frequency-independent, indicating the formation of a structural network from hardener particles [87]. Secondly, the storage modulus of the mixture exceeds its loss modulus in the region of low frequencies (long observation times), i.e., the system exhibits solid-like behavior. As the frequency becomes higher, the loss modulus of the mixture rises due to an increase in the loss modulus of the epoxy medium itself. In other words, the total viscoelasticity of the mixture is composed of the viscoelasticity of both the liquid epoxy matrix and the structural network of the hardener particles. Moreover, the properties of the particle network and the continuous medium determine principally the mixture’s behavior at low and high frequencies, respectively.

The addition of oil significantly affects the structure of the samples. When the concentration of one of the oils is 5–10%, the structural network from hardener particles breaks down. This destruction is accompanied by a decrease in the loss modulus in the low-frequency region and a drop in the storage modulus in the entire frequency range. In addition, coconut oil breaks the structure completely (the region of frequency-independent storage modulus disappears), while palm oil damages it only partially (since *G*′ ≈ *const* at least at low frequencies). Note that at a palm oil concentration of 20%, a new structure forms, giving the sample solid-like behavior. Palm oil is in a crystalline state at 25 °C, which probably leads to the appearance of a joint network structure of its particles with the hardener ones. The new structure has a higher storage modulus than the original sample, just like a higher yield stress (see Figure 3a). Meanwhile, 20% of coconut oil seems to cause wall slip and a drop in the measured moduli, which is probably due to the liquid state of this oil at the test temperature.

### 3.3. Effect of Oil Addition on Resin Curing

**Figure 5:**
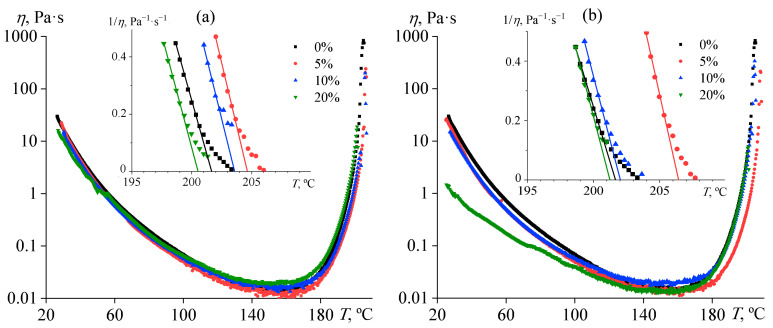
Viscosity changes during the curing of epoxy compositions containing palm oil (**a**) or coconut oil (**b**) in the regime of gradual temperature increase at a rate of 2 °C/min and a shear rate of 100 s^−1^. The oil concentration is indicated in the legends. The inserts show the determination of the gel points.

The general picture of viscosity changes when mixtures are smoothly heated is as follows. With increasing temperature, the effective viscosity of the mixtures gradually decreases firstly by three decimal orders of magnitude, coming to a minimum at 160 °C, and then starts to increase sharply due to the onset of the cross-linking process (Figure 5). As a result, the viscosity reaches a maximum at temperatures of 201–207 °C, accompanied by the detachment of the sample from the measuring surface of the rheometer. This temperature can be considered in the first approximation as the gel point. More accurately, it can be determined by extrapolating the inverse viscosity (1/*η*) to zero [88], as shown in the inserts in Figure 5.

The addition of vegetable oil introduces some changes in the rheokinetic of curing. When the concentration of coconut oil is 5–10%, the gelation process slows down. This result is most likely from a decrease in the concentration of reacting substances due to the dissolution of oil droplets in a continuous medium with an increase in temperature. The evaluation shows that the 5% concentration has the most substantial effect on the gelation: the gel point shifts towards high temperatures by about 5–7 °C. Meanwhile, palm oil in the same concentrations causes less significant changes, probably due to its worse solubility in the epoxy medium (see Figure 2).

The addition of 20% palm oil slightly speeds up cross-linking, which may be due to a decrease in the viscosity of the reaction mixture or dissolution of some of the epoxy resin in the oil droplets and increasing the relative content of the hardener in the continuous phase (pure hardener is insoluble in oils). In any case, this situation is possible according to the interferometry data: the solubility of palm oil is only about 12–16% at 160–180 °C, while the solubility of the resin in the oil is twice as high (35–40%, see Figure 2). In addition, an increase in temperature causes the disappearance of the wall slip that occurs in the case of 20% coconut oil. This result is consistent with interferometry data indicating that coconut oil is completely soluble in the resin medium at high temperatures. In other words, the heating decreases the concentration of dispersed coconut oil droplets, which gradually dissolve in a continuous medium as the temperature increases, and, accordingly, the wall slip effect disappears.

**Figure 6:**
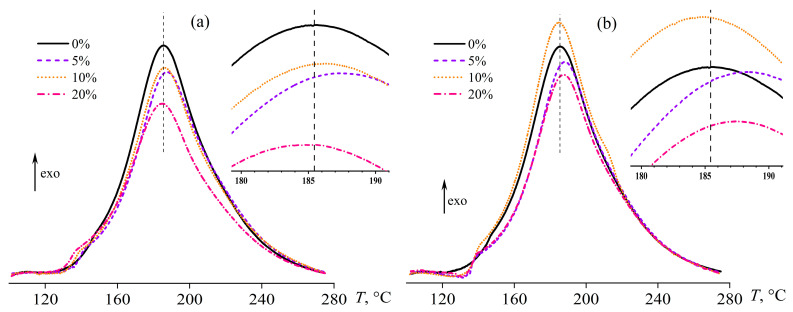
Changes in heat flow during the curing of epoxy compositions containing palm oil (**a**) or coconut oil (**b**) in the regime of gradual temperature increase at a rate of 2 °C/min. The inserts show an enlarged area near the heat release maximum.

Figure 6 shows heat flow thermograms for the samples containing palm or coconut oil in different percentages during their curing. The obtained curves show an exothermic peak that characterizes the cross-linking process of the resin. The temperature shift of the peak relative to the position that is specific for the oil-free blend (shown by the vertical dashed lines in Figure 6 and its inserts) can be used to evaluate the acceleration or deceleration of curing when oil is introduced. According to the thermograms, vegetable oils have little effect on the curing rate. Only 5% of coconut or palm oil noticeably shifts the heat release maximum toward higher temperatures (*T*_peak_, see Table 1), slowing down the curing, while the oil influence is less pronounced in other cases.

Thus, a comparison of calorimetry and rheometry data shows that 5% oil slows cross-linking in both tests, whereas 20% oil can accelerate gelation but does not change the cross-linking speed according to DSC. In the latter case, an explanation for the discrepancy may be in the different curing stages, whose rates are evaluated indirectly by these two methods. The gelation occurs due to the formation of spatial cross-links from the reaction of epoxy groups with secondary amino groups. This point is determined rheometrically. Meanwhile, the maximum on the DSC curves corresponds to lower temperatures, i.e., to a lower conversion degree of epoxy groups reacting predominantly with primary amino groups. In this respect, DSC is a less sensitive method for assessing the overall rate of epoxy curing, which includes reactions of epoxy groups with both primary and secondary amino groups.

Either of the two oils also slightly reduces the heat flow area (Δ*H*) because of the lowered concentration of the epoxy resin and the hardener in the total volume of the reacting mixture. However, when calculating the normalized heat effect of the curing (Δ*H*_r_ = Δ*H*/(1 − *c*_oil_/100)) with the account of the oil concentration (*c*_oil_), it is found that it is comparable for all of the systems within the measurement error (Table 1). In other words, the curing degree of the epoxy resin is not significantly affected by the introduction of oil.

### 3.4. Characteristics of Cured Epoxy Compositions

**Figure 7:**
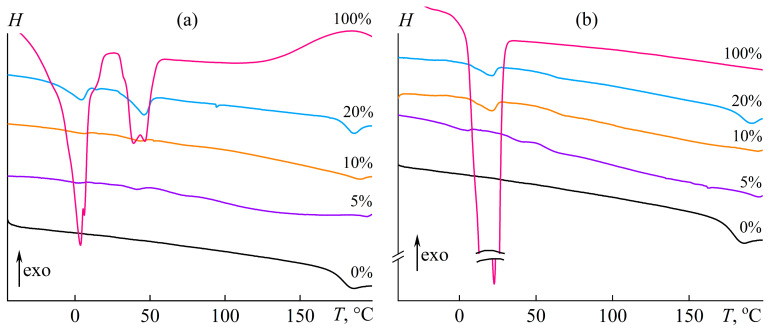
DSC thermograms of cured epoxy compositions containing palm oil (**a**) or coconut oil (**b**) with a weight fraction indicated near the curves.

Nevertheless, the oil introduction affects the thermophysical characteristics of the cured systems. Figure 7 shows DSC curves for pure vegetable oils and epoxy resin/hardener blends modified by them. Palm oil has two melting peaks (Figure 7a, 100%). The first peak corresponds to 3.7 °C and most likely results from unsaturated triglycerides in the oil composition. The second peak is 39.0 °C and probably relates to the melting of saturated triglycerides. In contrast, the thermogram for pure coconut oil has only one melting peak with a maximum of 22.7 °C (Figure 7b, 100%).

The introduction of 20% oil into the epoxy resin causes the appearance of one or two endothermic peaks in the case of coconut oil or palm oil, respectively. The position of the peaks coincides approximately with those of pure oils. Thus, these oils are dispersed at high concentrations and can crystallize in the cross-linked epoxy polymer. As the oil concentration decreases, the melting peak area decreases, and the peak position may shift slightly toward low or high temperatures (Table 2). However, it was found that the oils do not crystallize completely in the dispersed form. This situation may be due to the partial miscibility of these oils and epoxy resin, i.e., some oil compounds are dissolved in the cured polymer and do not participate in crystallization. Moreover, the epoxy resin can dissolve in the dispersed oil droplets (see Figure 2), which probably also suppresses oil crystallization.

Coconut oil showed better miscibility with epoxy resin than palm oil. As a result, its degree of crystallinity drops more significantly to 7–16% of the initial value (*DC*_oil_, see Table 2). The degree of crystallinity of palm oil is higher and reaches 17–65%. At the same time, there is no single dependence of the degree of crystallinity of the oil on its concentration. This fact is probably due to the variety of interrelated factors affecting oil crystallization, which include the size of oil droplets, the concentration of dissolved resin in the oil phase, and the concentration and composition of the oil dissolved in the cured polymer. The possibility of the latter factor is evidenced by the change in the glass transition temperature of the cured epoxy polymer.

On the one hand, the glass transition temperature, defined as the inflection point on the DSC curves, increases slightly with increasing oil concentration in the epoxy matrix. On the other hand, the glass transition range extends substantially toward lower temperatures, especially with low oil content, e.g., the glass transition starts at about 50 °C instead of 170 °C for the oil-free epoxy polymer. In this regard, vegetable oils plasticize the epoxy matrix despite the nominal increase in the glass transition temperature.

**Figure 8:**
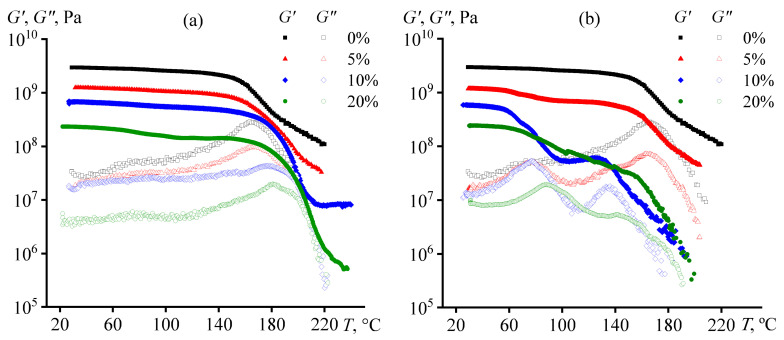
Temperature dependences of storage and loss moduli for epoxy compositions containing palm oil (**a**) or coconut oil (**b**). The oil weight fraction is given in the legends.

The effect of oil addition on the glass transition of the cured polymer can be traced more clearly using the dependences of its storage and loss moduli on temperature (Figure 8). With increasing temperature, the storage modulus of the oil-free epoxy matrix decreases, while the loss modulus increases up to the maximum and then decreases. In this case, the region of a sharp decrease in the storage modulus coincides with that where the loss modulus passes through the maximum. This temperature region is associated with the transition of the sample from the glass state to the rubbery state. The glass transition temperature can be determined from the location of the loss modulus maximum (165 °C for unmodified epoxy polymer) or from the point at which the storage modulus starts to decrease sharply (157 °C). The glass transition temperatures estimated by these two methods differ in magnitude because glass transition is a relaxation transition with a temperature that depends on determination conditions.

The storage and loss moduli decrease with increasing oil concentration in the epoxy composition, i.e., vegetable oils act as plasticizers of the epoxy polymer. In the case of samples containing coconut oil (which is better miscible with epoxy resin), the temperature dependencies of the loss modulus have two maxima (Figure 8b). This result means that these samples have two glass transition temperatures. The cause of this phenomenon may be a microphase separation of the mixture during the curing. One of the microphases is likely more enriched with vegetable oil, therefore having a lower concentration of the hardener (immiscible with oil), a lower cross-linking density, and consequently a lower glass transition temperature. The second microphase can be an oil-poor epoxy matrix with a higher glass transition temperature. Furthermore, the epoxy matrix consisting of two microphases also contains oil droplets released into a separate phase during the curing. The two-step transition upon heating from the glass to the rubber-like state initially of the first microphase and then of the second one leads to the storage modulus that seems to pass through a local maximum at 124 °C and 10% coconut oil mass fraction (Figure 8b). At this temperature, the oil-rich microphase is already in a rubber-like state, while the oil-poor one is still a glass. At 5% coconut oil content, the glass-to-rubber transition of the oil-rich microphase is practically invisible on the temperature dependence of the storage modulus, probably due to its low content. In turn, the 20% coconut oil causes a stretching of the storage modulus drop region over a wide temperature range, whereby the glass-to-rubbery transition of the oil-poor microphase is almost unnoticeable due to its reduced volume.

The picture of what happens during the curing of epoxy compositions containing palm oil is more straightforward. On the temperature dependencies of the loss modulus, there is only one maximum corresponding to the glass transition temperature (Figure 8a). In other words, there is no microphase separation and low-temperature glass transition. This result can be due to the worse solubility of palm oil in the epoxy resin when compared to coconut oil. As the oil concentration grows, the glass transition temperature increases (see Table 3, where the point of loss modulus maximum was assumed to be the glass transition temperature). Perhaps palm oil dissolving in the reaction medium at its low conversion rates contributes to more complete curing by facilitating the diffusion of the reacting substances. In our case, it is impossible to make reliable judgments about it since the heat release due to cross-linking changes within the measurement error upon oil addition (Δ*H*_r_, Table 1), while even a slight increase in the conversion of epoxy and amine groups leads to a substantial increase in the glass transition temperature of the epoxy polymer [89].

A similar increase in glass transition temperature with increasing oil content is observed for the coconut oil-rich microphase, i.e., the microphase with a lower glass transition temperature (Table 3). At the same time, coconut oil markedly reduces the value of the higher glass transition temperature compared to the glass transition temperature of the cured oil-free polymer. Furthermore, the storage modulus of the cured sample in the glass state decreases with the introduction of any of the oils and an increase in their concentration. Thus, the plasticization of the epoxy polymer occurs in all cases, while the higher the oil concentration, the greater this effect. In this case, the plasticizing impact is different for the storage modulus of the sample and its glass transition temperature. This situation is probably due to the complex concentration equilibrium in the resin/hardener/oil blend, which, in addition, changes as the molecular weight of the epoxy polymer increases because of its gradual cross-linking.

**Figure 9:**
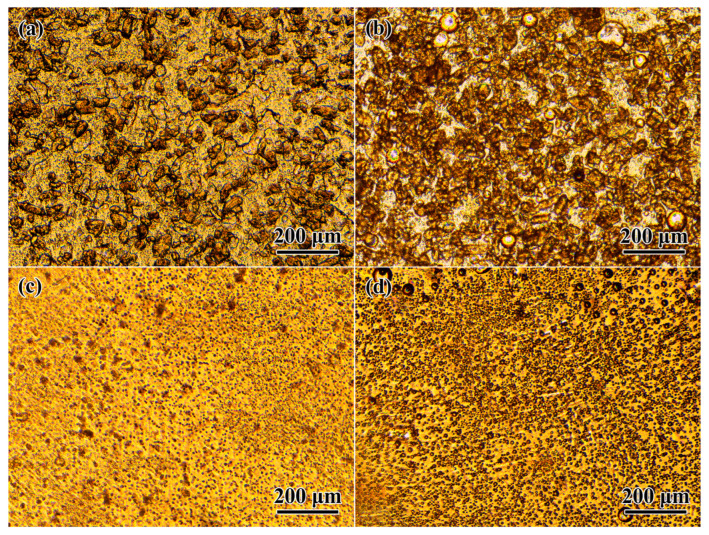
Microphotographs of epoxy resin/hardener mixtures containing 10% of palm oil (**a**,**c**) or coconut oil (**b**,**d**) before (**a**,**b**) and after the curing (**c**,**d**).

The curing of epoxy compositions results in the transformation of their structural and morphological features. According to microphotographs of uncured systems, there is a coalescence of oil droplets inside the epoxy medium with clearly visible agglomerates of hardener particles (Figure 9a,b). Given the low miscibility of vegetable oils and epoxy resin at 25 °C, there should be almost no mutual dissolution for them. However, the droplets of palm oil have shapes of extended areas, which may be due to the higher melting point of this oil and, consequently, its semi-solid consistency in the epoxy medium.

As a result of high-temperature curing at 180 °C, the hardener particles disappear, dissolving in the epoxy medium (Figure 9c,d). In addition, finely dispersed droplets replace extended oil areas and large oil droplets in the volume of the cured polymer. The epoxy resin is miscible with vegetable oils at high temperatures, i.e., there may be a temporary homogenization of the reacting system during its high-temperature curing with a subsequent phase separation because of growth in the epoxy molecular weight. In turn, the phase separation causes the formation of fine oil droplets evenly distributed in the volume of the cured matrix.

Palm oil produces smaller droplets, while coconut oil forms bigger and more numerous ones. This result is unusual because coconut oil is better miscible with the epoxy resin, and one would expect to obtain smaller droplets from this oil due to its more complete dissolution and the later loss of solubility during the curing. Nevertheless, the opposite result may still be because of the better miscibility, namely due to the partial dissolution of uncross-linked or weakly cross-linked epoxy resin in the forming coconut oil droplets during the phase separation of the system. As a result, the droplets are more numerous and grander in size, as they contain epoxy resin in addition to the oil. This conclusion is indirectly confirmed by DSC data, according to which coconut oil droplets have a lower degree of crystallinity (see Table 2), i.e., contain more dissolved epoxy resin that suppresses crystallization.

**Figure 10:**
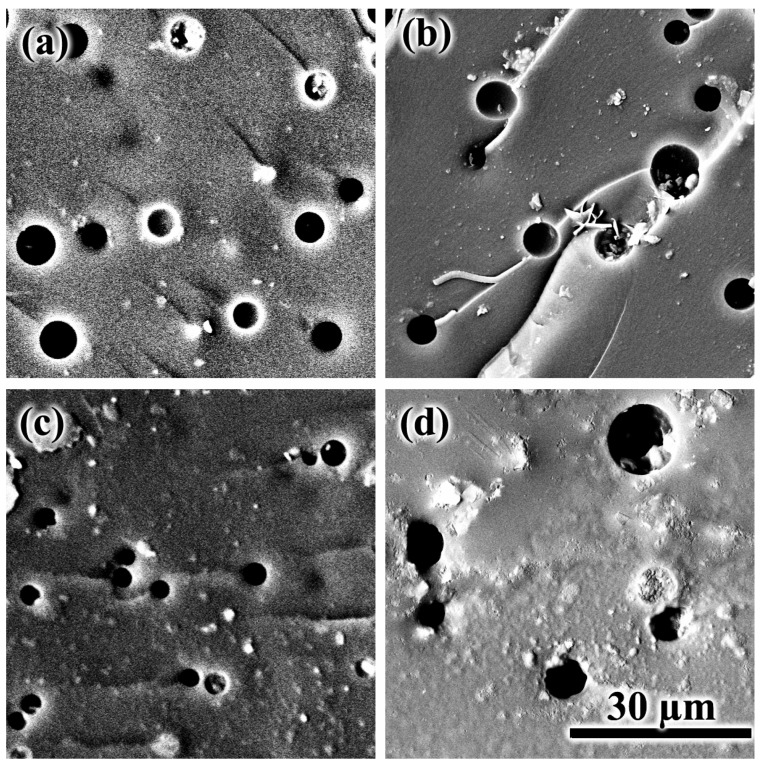
SEM images of the cross-section surface for epoxy resin cured at 180 °C and containing 5% (**a**,**c**) or 20% (**b**,**d**) palm oil (**a**,**b**) or coconut oil (**c**,**d**).

Electron microscopy allows for looking at the droplets in more detail (Figure 10). The droplets are random-distributed round-shaped voids, giving the sample a porous structure in appearance. If we compare systems with 5% oil content, palm oil forms larger droplets (7–8 µm) than coconut oil (3 µm). These oils are fully miscible at this content with the curing epoxy matrix regardless of their type. Thus, the smaller size of the coconut oil droplets may indicate a later phase separation, which occurs in a general case due to an increase in the molecular weight of the reacting epoxy resin and a decrease in its miscibility with the oil. Because of the later phase separation, the oil droplets have less time to grow and merge, and the rates of these processes are also inhibited by the higher viscosity of the epoxy medium due to its higher degree of curing. Indeed, coconut oil is better miscible with the epoxy resin, meaning that we can expect a longer residence time of the reacting system in a homogeneous state and, consequently, a smaller size of the resulting droplets in its case.

Higher oil concentrations should shift the phase separation point toward lower degrees of curing and thus result in larger droplet sizes due to longer droplet growth and coalescence. Indeed, the increase in coconut oil content from 5% to 20% leads to a marked increase in droplet diameter from 3 μm to 14 μm (Figure 10c,d). At the same time, the droplet size of palm oil practically does not change with the same increase in its concentration, remaining at 7–8 μm (Figure 10a,b), probably due to a lesser influence of the concentration of this oil on the phase separation point.

The specific heat storage capacity of the obtained phase-change materials is relatively small in terms of the practical aspect of their application. Firstly, this situation is due to the limit of the maximum mass fraction of oil droplets in the epoxy matrix at 20%. Secondly, it is the suppression of oil crystallization, especially in the case of coconut oil, down to 7.3% from the initial level (see Table 2). These problems necessitate exploring an alternative application of the obtained oil-containing materials. Their advantage is the absence of significant plasticizing of the cured epoxy matrix by vegetable oils, which helps to maintain its high stiffness and glass transition temperature. In addition, the surface of these materials is oily, and vegetable and other ester oils are used widely as the base oils for greases [90,91,92]. These facts suggest the utilization of the obtained oil-containing materials as antifriction ones. Epoxy plastics can be materials for manufacturing bearing cages and separators [93,94,95,96], which should have a low friction coefficient and high wear resistance (i.e., high hardness and stiffness), and the obtained materials seem to meet these requirements. Moreover, the oil-containing materials are self-lubricating due to (1) the oil diffusion onto their surface and (2) its automatic oiling when it wears with a release of dispersed oil droplets. The self-lubricating property reduces noise during friction and eliminates the need for lubricant supply to the bearing, which was noticeable when tribotesting the cured oil-containing materials even after their preliminary degreasing with acetone.

**Figure 11:**
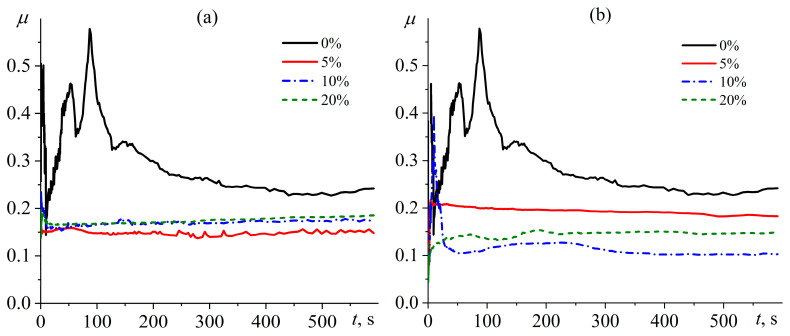
Time dependences of friction coefficient for epoxy compositions containing palm oil (**a**) or coconut oil (**b**) during their dry friction on steel. The oil weight fraction is given in the legends.

**Figure 12:**
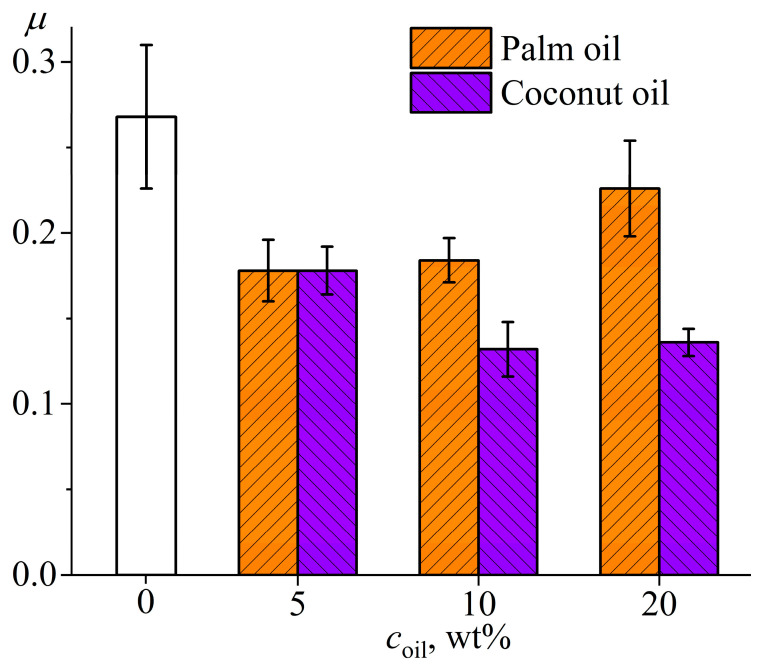
The steady-state friction coefficient for epoxy compositions containing different mass fractions of vegetable oils during their dry friction on steel.

Figure 11 shows the dependence of friction coefficient on time during high-speed friction of the rotating steel plate on surfaces of different cured epoxy compositions. The oil-free epoxy plastic laps to the rotating steel plate within the first 5 min, causing noticeable noise and instability of the friction coefficient. After lapping, the friction coefficient reaches a steady state of about 0.27 ± 0.04. The tribological behavior of oil-containing plastics is fundamentally different. In all cases, the oil within the epoxy polymer leads to the absence of a noticeable lapping zone, noiseless friction, and a significant reduction in the friction coefficient. Depending on the formulation of the oil-containing compositions, the friction coefficient changes from 0.13 to 0.23 (Figure 12). The increase in the mass fraction of palm oil from 5% to 20% raises the friction coefficient from 0.18 to 0.23, which can be due to the semi-crystalline state of this oil at 25 °C and increased degree of its crystallinity at higher concentrations (see Table 2). Conversely, an increase in the mass content of coconut oil from 5% to 10–20% reduces the friction coefficient from 0.18 to 0.13–0.14, which can be due to an increase in oil concentration and its liquid state at the temperature of the tribological tests. After these tests, there are no visible signs of wear, unlike the oil-free plastic with marks from lapping.

## 4. Conclusions

Under normal temperature conditions, an epoxy resin modified with a high-melting vegetable oil can be a homogeneous solution, but only if the oil content is low at about 1–3%. At higher oil concentrations in the epoxy medium, emulsions form with oil droplets containing about 8–11% dissolved epoxy resin. When heated, the miscibility of the oil and epoxy resin increases, allowing for temporary homogenizing of the system during its curing process. As cross-linking proceeds, the molecular weight of the epoxy polymer increases, leading to phase separation with the formation of finely dispersed oil droplets. These droplets retain their ability to crystallize and thus give the cross-linked epoxy polymer the properties of a phase-change material. The advantage of this method is the production of emulsion systems without the use of surfactants and the shape stability of the resulting PCM. At the same time, it has some drawbacks. Firstly, because of the limited miscibility between the oil and the epoxy resin, some of the oil remains dissolved in the cured epoxy polymer and thus reduces its elastic modulus and expands the glass transition region towards low temperatures. Secondly, due to the same limited miscibility, some of the epoxy resin remains dissolved in the oil droplets and thus reduces their degree of crystallinity, lowering the energy efficiency of the resulting phase-change material. Thirdly, the maximum oil content in the epoxy resin is limited to 20–40% because of either the limited oil miscibility even at elevated temperatures or the formation of resin-in-oil emulsions at higher oil concentrations. These problems may be solved by selecting a specific epoxy resin, hardener, and high-melting oil. In the case of diglycidyl ether of bisphenol A, 4,4’-diaminodiphenyl sulfone, and vegetable oils, palm oil allows for storing its crystallization heat better. Potential applications for such epoxy heat accumulators are possible in home construction in the production of smart energy-saving epoxy floors combined with underfloor heating systems: during the warm day, these floors accumulate thermal energy, while at night, they release it back, staying warmer and reducing the energy consumption for their heating. An alternative application is self-lubricating materials for creating low-friction bearing separators with reduced noise and no need for external lubrication. At the same time, palm oil is better suited for creating phase-change materials due to the better crystallization of its dispersed droplets, while coconut oil is unsuitable for creating phase-change materials due to suppressed crystallization but serves better for obtaining self-lubricating materials with a lower friction coefficient.

## Figures and Tables

**Table 1:** Thermophysical features of epoxy curing.

Oil Type	*c*_oil_, wt.%	*T*_peak_, °C	Δ*H*, J/g	Δ*H*_r_, J/g
w/o	0	185.6	417	417
Palm	5	187.4	379	399
Palm	10	186.2	392	436
Palm	20	185.5	337	421
Coconut	5	188.3	396	417
Coconut	10	184.9	390	434
Coconut	20	185.5	337	421
Standard deviation	±0.01 wt.%	±0.2 °C	±21 J/g	±21 J/g

**Table 2:** Thermophysical characteristics of cured epoxy compositions.

Oil Type	*c*_oil_, wt.%	*T*_g,DSC_, °C	∆*H*_m1_, J/g	*T*_m1_*,* °C	∆*H*_m2_, J/g	*T*_m2_*,* °C	*DC*_oil_, %
w/o	0	178.1	–	–	–	–	–
Palm	5	190.9	0.34	0.6	0.37	40.8	29.0
Palm	10	186.7	0.35	4.1	0.46	42.5	16.6
Palm	20	181.1	2.72	4.1	3.67	45.8	65.3
Palm	100	–	48.9	3.7	20.7	39.0	100
Coconut	5	190.9	0.48	1.9	0.31	38.8	15.8
Coconut	10	191.3	1.39	20.0	–	–	13.9
Coconut	20	185.0	1.45	21.1	–	–	7.3
Coconut	100	–	99.7	22.7	–	–	100
Standard deviation	±0.01 wt.%	±0.2 °C	±5% *	±0.2 °C	±5% *	±0.2	±5% *

* A relative error is specified.

**Table 3:** Storage modulus and glass transition temperature of cured epoxy compositions.

Oil Type	*c*_oil_, wt.%	*T*_g,DMA_, °C	*G*′_25°C_, GPa
w/o	0	165	2.96
Palm	5	166	1.25
Palm	10	175	0.68
Palm	20	180	0.23
Coconut	5	77 and 163	1.18
Coconut	10	76 and 135	0.59
Coconut	20	88 and 140	0.24
Standard deviation	±0.01 wt.%	±1 °C	±5% *

* A relative error is specified.

## Data Availability

The data presented in this study are available on request from the corresponding author.

## References

[1] Panwar N.L., Kaushik S.C., Kothari S. (2011). Role of renewable energy sources in environmental protection: A review. Renew. Sustain. Energy Rev..

[2] Sarbu I., Sebarchievici C. (2018). A comprehensive review of thermal energy storage. Sustainability.

[3] Solomon B.D., Krishna K. (2011). The coming sustainable energy transition: History, strategies, and outlook. Energy Policy.

[4] Navarro M.E., Martínez M., Gil A., Fernández A.I., Cabeza L.F., Olives R., Py X. (2012). Selection and characterization of recycled materials for sensible thermal energy storage. Sol. Energy Mater. Sol. Cells.

[5] Zhou D., Zhao C.Y., Tian Y. (2012). Review on thermal energy storage with phase change materials (PCMs) in building applications. Appl. Energy.

[6] Raoux S. (2009). Phase Change Materials. Annu. Rev. Mater. Res..

[7] Demirbas M.F. (2006). Thermal Energy Storage and Phase Change Materials: An Overview. Energy Sources Part B Econ. Plan. Policy.

[8] Sharma A., Tyagi V.V., Chen C.R., Buddhi D. (2009). Review on thermal energy storage with phase change materials and applications. Renew. Sustain. Energy Rev..

[9] Farid M.M., Khudhair A.M., Razack S.A.K., Al-Hallaj S. (2004). A review on phase change energy storage: Materials and applications. Energy Convers. Manag..

[10] Kalnæs S.E., Jelle B.P. (2015). Phase change materials and products for building applications: A state-of-the-art review and future research opportunities. Energy Build..

[11] Gorbacheva S.N., Makarova V.V., Ilyin S.O. (2021). Hydrophobic nanosilica-stabilized graphite particles for improving thermal conductivity of paraffin wax-based phase-change materials. J. Energy Storage.

[12] Sarier N., Onder E. (2012). Organic phase change materials and their textile applications: An overview. Thermochim. Acta.

[13] Kenisarin M.M. (2014). Thermophysical properties of some organic phase change materials for latent heat storage. A review. Sol. Energy.

[14] Zhang P., Xiao X., Ma Z.W. (2016). A review of the composite phase change materials: Fabrication, characterization, mathematical modeling and application to performance enhancement. Appl. Energy.

[15] Makarova V.V., Gorbacheva S.N., Antonov S.V., Ilyin S.O. (2020). On the Possibility of a Radical Increase in Thermal Conductivity by Dispersed Particles. Russ. J. Appl. Chem..

[16] Lin Y., Alva G., Fang G. (2018). Review on thermal performances and applications of thermal energy storage systems with inorganic phase change materials. Energy.

[17] Gorbacheva S.N., Ilyin S.O. (2021). Structure, rheology and possible application of water-in-oil emulsions stabilized by asphaltenes. Colloids Surf. A Physicochem. Eng. Asp..

[18] Mohamed S.A., Al-Sulaiman F.A., Ibrahim N.I., Zahir M.H., Al-Ahmed A., Saidur R., Yılbaş B.S., Sahin A.Z. (2017). A review on current status and challenges of inorganic phase change materials for thermal energy storage systems. Renew. Sustain. Energy Rev..

[19] Rathod M.K., Banerjee J. (2013). Thermal stability of phase change materials used in latent heat energy storage systems: A review. Renew. Sustain. Energy Rev..

[20] Kahwaji S., White M.A. (2018). Prediction of the properties of eutectic fatty acid phase change materials. Thermochim. Acta.

[21] Vasilyev G., Koifman N., Shuster M., Gishvoliner M., Cohen Y., Zussman E. (2020). Synergistic Effect of Two Organogelators for the Creation of Bio-Based, Shape-Stable Phase-Change Materials. Langmuir.

[22] Pielichowska K., Pielichowski K. (2014). Phase change materials for thermal energy storage. Prog. Mater. Sci..

[23] Chen C., Liu W., Wang Z., Peng K., Pan W., Xie Q. (2015). Novel form stable phase change materials based on the composites of polyethylene glycol/polymeric solid-solid phase change material. Sol. Energy Mater. Sol. Cells.

[24] Jin Z., Wang Y., Liu J., Yang Z. (2008). Synthesis and properties of paraffin capsules as phase change materials. Polymer.

[25] Zhu Y., Qin Y., Wei C., Liang S., Luo X., Wang J., Zhang L. (2018). Nanoencapsulated phase change materials with polymer-SiO_2_ hybrid shell materials: Compositions, morphologies, and properties. Energy Convers. Manag..

[26] Tahan Latibari S., Mehrali M., Mehrali M., Indra Mahlia T.M., Cornelis Metselaar H.S. (2013). Synthesis, characterization and thermal properties of nanoencapsulated phase change materials via sol–gel method. Energy.

[27] Alehosseini E., Jafari S.M. (2019). Micro/nano-encapsulated phase change materials (PCMs) as emerging materials for the food industry. Trends Food Sci. Technol..

[28] Fang Y., Kuang S., Gao X., Zhang Z. (2008). Preparation and characterization of novel nanoencapsulated phase change materials. Energy Convers. Manag..

[29] Liu C., Rao Z., Zhao J., Huo Y., Li Y. (2015). Review on nanoencapsulated phase change materials: Preparation, characterization and heat transfer enhancement. Nano Energy.

[30] Su W., Darkwa J., Kokogiannakis G. (2015). Review of solid–liquid phase change materials and their encapsulation technologies. Renew. Sustain. Energy Rev..

[31] Lian Q., Li K., Sayyed A.A.S., Cheng J., Zhang J. (2017). Study on a reliable epoxy-based phase change material: Facile preparation, tunable properties, and phase/microphase separation behavior. J. Mater. Chem. A.

[32] Weingrill H.M., Resch-Fauster K., Zauner C. (2018). Applicability of Polymeric Materials as Phase Change Materials. Macromol. Mater. Eng..

[33] Grace H.P. (1982). Dispersion phenomena in high viscosity immiscible fluid systems and application of static mixers as dispersion devices in such systems. Chem. Eng. Commun..

[34] Gorbacheva S.N., Ilyin S.O. (2021). Morphology and Rheology of Heavy Crude Oil/Water Emulsions Stabilized by Microfibrillated Cellulose. Energy Fuels.

[35] Vinogradov G.V. (1977). Ultimate regimes of deformation of linear flexible chain fluid polymers. Polymer.

[36] Vinogradov G.V., Yanovsky Y.G., Titkova L.V., Barancheeva V.V., Sergeenkov S.I., Borisenkova E.K. (1980). Viscoelastic properties of linear polymers in the fluid state and their transition to the high-elastic state. Polym. Eng. Sci..

[37] Kostyuk A.V., Smirnova N.M., Ilyin S.O. (2022). Two-functional phase-change pressure-sensitive adhesives based on polyisobutylene matrix filled with paraffin wax. J. Energy Storage.

[38] Wang Z., Situ W., Li X., Zhang G., Huang Z., Yuan W., Yang C. (2017). Novel shape stabilized phase change material based on epoxy matrix with ultrahigh cycle life for thermal energy storage. Appl. Therm. Eng..

[39] Ma T., Li L., Wang Q., Guo C. (2019). High-performance flame retarded paraffin/epoxy resin form-stable phase change material. J. Mater. Sci..

[40] Ignatenko V.Y., Ilyin S.O., Kostyuk A.V., Bondarenko G.N., Antonov S.V. (2020). Acceleration of epoxy resin curing by using a combination of aliphatic and aromatic amines. Polym. Bull..

[41] Amberkar T., Mahanwar P. (2023). Thermal Energy Management in Buildings and Constructions with Phase Change Material-Epoxy Composites: A Review. Energy Sources Part A Recovery Util. Environ. Eff..

[42] Ren X., Shen H., Yang Y., Yang J. (2019). Study on the Properties of a Novel Shape-Stable Epoxy Resin Sealed Expanded Graphite/Paraffin Composite PCM and Its Application in Buildings. Phase Transit..

[43] Luo W., Hu X., Che Y., Zu S., Li Q., Jiang X., Liu D. (2022). Form-Stable Phase Change Materials Enhanced Photothermic Conversion and Thermal Conductivity by Ag-Expanded Graphite. J. Energy Storage.

[44] Ilyina S.O., Vlasova A.V., Gorbunova I.Y., Lukashov N.I., Kerber M.L., Ilyin S.O. (2023). Epoxy Phase-Change Materials Based on Paraffin Wax Stabilized by Asphaltenes. Polymers.

[45] Ohayon-Lavi A., Lavi A., Alatawna A., Ruse E., Ziskind G., Regev O. (2021). Graphite-Based Shape-Stabilized Composites for Phase Change Material Applications. Renew. Energy.

[46] Wu B., Jiang Y., Wang Y., Zhou C., Zhang X., Lei J. (2018). Study on a PEG/Epoxy Shape-Stabilized Phase Change Material: Preparation, Thermal Properties and Thermal Storage Performance. Int. J. Heat Mass Transf..

[47] Lian Q., Li Y., Sayyed A.A.S., Cheng J., Zhang J. (2018). Facile Strategy in Designing Epoxy/Paraffin Multiple Phase Change Materials for Thermal Energy Storage Applications. ACS Sustain. Chem. Eng..

[48] Arinina M.P., Ilyin S.O., Makarova V.V., Gorbunova I.Y., Kerber M.L., Kulichikhin V.G. (2015). Miscibility and rheological properties of epoxy resin blends with aromatic polyethers. Polym. Sci. Ser. A.

[49] Shapagin A.V., Budylin N.Y., Chalykh A.E., Solodilov V.I., Korokhin R.A., Poteryaev A.A. (2021). Phase Equilibrium, Morphology, and Physico-Mechanics in Epoxy-Thermoplastic Mixtures with Upper and Lower Critical Solution Temperatures. Polymers.

[50] Ignatenko V.Y., Kostyuk A.V., Kostina J.V., Bakhtin D.S., Makarova V.V., Antonov S.V., Ilyin S.O. (2020). Heavy crude oil asphaltenes as a nanofiller for epoxy resin. Polym. Eng. Sci..

[51] Kim J., Kim D.N., Lee S.H., Yoo S.H., Lee S. (2010). Correlation of fatty acid composition of vegetable oils with rheological behaviour and oil uptake. Food Chem..

[52] Volkov V.P., Roginskaya G.F., Chalykh A.E., Rozenberg B.A. (1982). Phase Structure of Epoxide–Rubber Systems. Russ. Chem. Rev..

[53] Chalykh A.E., Gerasimov V.K. (2004). Phase equilibria and phase structures of polymer blends. Russ. Chem. Rev..

[54] Ilyin S.O., Makarova V.V., Polyakova M.Y., Kulichikhin V.G. (2020). Phase state and rheology of polyisobutylene blends with silicone resin. Rheol. Acta.

[55] Ilyin S.O., Makarova V.V., Polyakova M.Y., Kulichikhin V.G. (2020). Phase behavior and rheology of miscible and immiscible blends of linear and hyperbranched siloxane macromolecules. Mater. Today Commun..

[56] Qi B., Zhang Q., Sui X., Wang Z., Li Y., Jiang L. (2016). Differential scanning calorimetry study–assessing the influence of composition of vegetable oils on oxidation. Food Chem..

[57] Kratzeisen M., Müller J. (2010). Influence of free fatty acid content of coconut oil on deposit and performance of plant oil pressure stoves. Fuel.

[58] Ilyin S.O., Yadykova A.Y., Makarova V.V., Yashchenko V.S., Matveenko Y.V. (2020). Sulfonated polyoxadiazole synthesis and processing into ion-conducting films. Polym. Int..

[59] Ilyin S.O., Kulichikhin V.G. (2023). Rheology and Miscibility of Linear/Hyperbranched Polydimethylsiloxane Blends and an Abnormal Decrease in Their Viscosity. Macromolecules.

[60] Schramm G. (2004). A Practical Approach to Rheology and Rheometry.

[61] Yadykova A.Y., Ilyin S.O. (2022). Rheological and Adhesive Properties of Nanocomposite Bitumen Binders Based on Hydrophilic or Hydrophobic Silica and Modified with Bio-Oil. Constr. Build. Mater..

[62] Scott R.L. (1949). The thermodynamics of high polymer solutions. IV. Phase equilibria in the ternary system: Polymer–liquid 1–liquid 2. J. Chem. Phys..

[63] Ilyin S.O., Makarova V.V., Anokhina T.S., Ignatenko V.Y., Brantseva T.V., Volkov A.V., Antonov S.V. (2018). Diffusion and phase separation at the morphology formation of cellulose membranes by regeneration from N-methylmorpholine N-oxide solutions. Cellulose.

[64] Minton A.P. (2020). Simple Calculation of Phase Diagrams for Liquid-Liquid Phase Separation in Solutions of Two Macromolecular Solute Species. J. Phys. Chem. B.

[65] Hansen C.M. (2007). Hansen Solubility Parameters: A User’s Handbook.

[66] Hoy K.L. (1989). Solubility Parameter as a Design Parameter for Water Borne Polymers and Coatings. J. Coat. Fabr..

[67] Barton A.F.M. (1990). Handbook of Polymer-Liquid Interaction Parameters and Solubility Parameters.

[68] Cepeda E.A., Bravo R., Lomas J.M. (2012). Solubilities of Fatty Acids and Triglycerides in 1-Bromopropane. J. Chem. Eng. Data.

[69] MacKnight W.J., Karasz F.E. (1989). Polymer Blends. Comprehensive Polymer Science and Supplements.

[70] Chung T.S. (1997). The limitations of using Flory-Huggins equation for the states of solutions during asymmetric hollow-fiber formation. J. Membr. Sci..

[71] Vinogradov G.V., Insarova N.I., Boiko B.B., Borisenkova E.K. (1972). Critical regimes of shear in linear polymers. Polym. Eng. Sci..

[72] Borisenkova E.K., Dreval V.E., Vinogradov G.V., Kurbanaliev M.K., Moiseyev V.V., Shalganova V.G. (1982). Transition of polymers from the fluid to the forced high-elastic and leathery states at temperatures above the glass transition temperature. Polymer.

[73] Yadykova A.Y., Strelets L.A., Ilyin S.O. (2023). Infrared Spectral Classification of Natural Bitumens for Their Rheological and Thermophysical Characterization. Molecules.

[74] Ilyin S.O., Ignatenko V.Y., Kostyuk A.V., Levin I.S., Bondarenko G.N. (2022). Deasphalting of heavy crude oil by hexamethyldisiloxane: The effect of a solvent/oil ratio on the structure, composition, and properties of precipitated asphaltenes. J. Pet. Sci. Eng..

[75] Brantseva T.V., Ilyin S.O., Gorbunova I.Y., Antonov S.V., Korolev Y.M., Kerber M.L. (2016). Epoxy reinforcement with silicate particles: Rheological and adhesive properties-Part II: Characterization of composites with halloysite. Int. J. Adhes. Adhes..

[76] Ilyin S.O., Arinina M.P., Malkin A.Y., Kulichikhin V.G. (2016). Sol–gel transition and rheological properties of silica nanoparticle dispersions. Colloid J..

[77] Chow M.C., Ho C.C. (2000). Surface active properties of palm oil with respect to the processing of palm oil. J. Oil Palm Res..

[78] Blijdenstein T.B., van der Linden E., van Vliet T., van Aken G.A. (2004). Scaling behavior of delayed demixing, rheology, and microstructure of emulsions flocculated by depletion and bridging. Langmuir.

[79] Datta S.S., Gerrard D.D., Rhodes T.S., Mason T.G., Weitz D.A. (2011). Rheology of attractive emulsions. Phys. Rev. E.

[80] Mironova M.V., Ilyin S.O. (2018). Effect of silica and clay minerals on rheology of heavy crude oil emulsions. Fuel.

[81] Yadykova A.Y., Ilyin S.O. (2021). Rheological, thermophysical, and morphological features of original and hydrogenated bio-oils. Sustain. Energy Fuels.

[82] Sánchez M.C., Valencia C., Franco J.M., Gallegos C. (2001). Wall slip phenomena in oil-in-water emulsions: Effect of some structural parameters. J. Colloid Interface Sci..

[83] Ilyin S.O., Malkin A.Y., Kulichikhin V.G., Shaulov A.Y., Stegno E.V., Berlin A.A., Patlazhan S.A. (2014). Rheological properties of polyethylene/metaboric acid thermoplastic blends. Rheol. Acta.

[84] Zhang X., Lorenceau E., Bourouina T., Basset P., Oerther T., Ferrari M., Coussot P. (2018). Wall slip mechanisms in direct and inverse emulsions. J. Rheol..

[85] Ilyin S.O., Kulichikhin V.G., Malkin A.Y. (2015). Rheological properties of emulsions formed by polymer solutions and modified by nanoparticles. Colloid Polym. Sci..

[86] Kostyuk A.V., Ignatenko V.Y., Makarova V.V., Antonov S.V., Ilyin S.O. (2020). Polyethylene Wax as an Alternative to Mineral Fillers for Preparation of Reinforced Pressure-Sensitive Adhesives. Int. J. Adhes. Adhes..

[87] Malkin A.Y., Ilyin S.O., Arinina M.P., Kulichikhin V.G. (2017). The rheological state of suspensions in varying the surface area of nano-silica particles and molecular weight of the poly(ethylene oxide) matrix. Colloid Polym. Sci..

[88] Arinina M.P., Kostenko V.A., Gorbunova I.Y., Il’in S.O., Malkin A.Y. (2018). Kinetics of curing of epoxy resin by diaminodiphenyl sulfone: Rheology and calorimetry. Polym. Sci. Ser. A.

[89] Galy J., Sabra A., Pascault J.P. (1986). Characterization of epoxy thermosetting systems by differential scanning calorimetry. Polym. Eng. Sci..

[90] Martín-Alfonso J.E., López-Beltrán F., Valencia C., Franco J.M. (2018). Effect of an Alkali Treatment on the Development of Cellulose Pulp-Based Gel-like Dispersions in Vegetable Oil for Use as Lubricants. Tribol. Int..

[91] Ilyin S.O., Gorbacheva S.N., Yadykova A.Y. (2023). Rheology and Tribology of Nanocellulose-Based Biodegradable Greases: Wear and Friction Protection Mechanisms of Cellulose Microfibrils. Tribol. Int..

[92] Hamnas A., Unnikrishnan G. (2023). Bio-Lubricants from Vegetable Oils: Characterization, Modifications, Applications and Challenges—Review. Renew. Sustain. Energy Rev..

[93] Scott D., Blackwell J., McCullagh P.J., Mills G.H. (1970). Composite Materials for Rolling Bearing Cages. Wear.

[94] Feyzullahoglu E., Saffak Z. (2008). The Tribological Behaviour of Different Engineering Plastics under Dry Friction Conditions. Mater. Des..

[95] Friedrich K. (2018). Polymer Composites for Tribological Applications. Adv. Ind. Eng. Polym. Res..

[96] Bu Y., Xu M., Liang H., Gao K., Zhang Y., Chen B., Min C., Hua X., Fu Y. (2021). Fabrication of Low Friction and Wear Carbon/Epoxy Nanocomposites Using the Confinement and Self-Lubricating Function of Carbon Nanocage Fillers. Appl. Surf. Sci..

